# Machine learning for Charcot-Marie-Tooth disease: toward predictive, personalized neuropathy care

**DOI:** 10.1097/MS9.0000000000005154

**Published:** 2026-05-13

**Authors:** Alara Apa, Falak Naz, Muhammad Talha, Sakan Binte Imran, Laiba Shamim

**Affiliations:** a Department of Medicine, Acıbadem University School of Medicine, Istanbul Türkiye; b Department of Medicine, Chandka Medical College Larkana, Sindh, Pakistan; c Department of Medicine, King Edward Medical University, Lahore, Pakistan; d Department of Medicine, Sir Salimullah Medical College, Dhaka, Bangladesh; e Department of Medicine, Jinnah Sindh Medical University, Karachi, Pakistan

*To the Editor,*

Charcot-Marie-Tooth disease (CMT) is a hereditary neuropathy with several complications that impact the motor and sensory nerves, significantly increasing the morbidity of the disease. Although no treatment changes the disease course, the management process has multiple parts that, if implemented according to the established guidelines, can help improve quality of life and lead to a delay in complications, especially if executed at the early stages of the disease^[^1^]^. Currently, many machine learning (ML) models have achieved great success in the detection of subclinical neuropathy, which could help provide significant value in the early diagnosis of CMT.

ML models, including deep neural networks, support vector machines, K-nearest neighbor, logistic regression, and random forest, have all shown promising ability to accurately predict and detect nerve damage early. A deep neural network algorithm developed to improve nerve conduction velocity (NCV) analysis was able to identify subclinical neuropathy with 92% sensitivity 6 months ahead of the standard methods^[^2^]^. In another study investigating the accuracy of several ML models to detect early nerve damage through nerve conduction studies to achieve a timely diagnosis of Guillain-Barre Syndrome, it was found that logistic regression and random forest models provided the best accuracy at 93%^[^3^]^. Implementing ML models for the detection of subclinical neuropathy in a clinical setting can improve the management process of CMT disease.

Current studies specifically targeting CMT have revealed the promising intersection of electrophysiology, imaging, and ML. High-resolution ultrasound has repeatedly shown significantly increased nerve cross-sectional area (CSA) in both adults and children with CMT, up to 3.5-fold compared to controls, with CSA strongly correlating with disease severity in CMT1A patients (e.g., higher CMTNS2 scores)^[^4^]^. A large meta-analysis encompassing over 6000 nerve measurements confirmed diffuse nerve enlargement across multiple genetic CMT types, providing a rich substrate of imaging biomarkers for ML algorithms^[^5^]^. Furthermore, studies combining shear wave elastography and NCV revealed that nerve stiffness and CSA were positively correlated and inversely correlated with motor nerve conduction velocity in CMT1A^[^6^]^. These imaging and electrophysiological databases lay the groundwork for ML systems capable of detecting and stratifying subclinical nerve damage in CMT patients.

Translating these insights into clinical ML tools for CMT faces several hurdles. First, data scarcity and heterogeneity may complicate data integration and model training, as thousands of CSA measurements exist, which are derived from diverse cohorts, imaging protocols, age groups, and CMT subtypes^[^5^]^. Second, feature instability poses a threat – models tuned to particular ultrasound metrics may underperform when faced with data acquired on different ultrasound machines or by different operators. Third, interpretability remains critical because clinicians require insight into how ML models reach their conclusions; black-box outputs without clear reasoning are unlikely to inspire trust, particularly in neurological diagnostics. Finally, clinical integration challenges, such as differences in hospital IT systems, workflow disruption, data privacy laws (e.g., GDPR and HIPAA), and the need for prospective validation, further limit the real-world deployment of ML-based tools^[^1,5^]^. Unless these issues are systematically addressed, ML algorithms may remain confined to research.

ML holds a significant role in the early detection and improved outcomes of subclinical neuropathy in CMT disease by using imaging and electrophysiological data. However, clinical implementation requires overcoming challenges, including data variability, lack of standardized protocols, and limited model interpretability. Further efforts should focus on building multicenter datasets, developing explainable AI models, and conducting real-world validation studies. Incorporation into clinical practice must also consider regulatory and infrastructural capacity. With these steps, ML can transition from experimental use to a practical tool in personalized CMT care.

## Data Availability

Not applicable.
